# Pelvic girdle pain affects the whole life—a qualitative interview study in Norway on women’s experiences with pelvic girdle pain after delivery

**DOI:** 10.1186/1756-0500-7-686

**Published:** 2014-10-03

**Authors:** Jorun Engeset, Britt Stuge, Liv Fegran

**Affiliations:** Department of Physical Medicine and Rehabilitation, Sørlandet Hospital, Post Box 416, NO-4604 Kristiansand, Norway; Department of Orthopaedics, Oslo University Hospital, Kirkeveien 166, NO-0407 Oslo, Norway; Faculty of Health and Sport Sciences, University of Agder, Post Box 422, NO-4604 Kristiansand, Norway; Sørlandet Hospital Research Unit, Post Box 416, NO-4604 Kristiansand, Norway

**Keywords:** Pelvic girdle pain, Post-partum, Phenomenology, Hermeneutics

## Abstract

**Background:**

The aim of this study was to explore how pelvic girdle pain after delivery influences women’s daily life in Norway. Knowledge about living with post-partum pelvic girdle pain is lacking.

**Method:**

A phenomenological–hermeneutical design with qualitative semi-structured interviews was used. A strategic selection procedure was chosen to recruit participants from physiotherapy clinics and a regional hospital in Norway. Five women with clinically verified pelvic girdle pain after delivery were included. Data were imported into NVivo9 and analysed in three steps: naïve reading, structural analysis and comprehensive understanding of the text.

**Results:**

Three themes influencing the women’s daily life were identified: 1) activity and pain, 2) lack of acknowledgment of pain and disability, and 3) changed roles. A daily life with pain and limited physical activity was difficult to accept and made some of the women feel discouraged, isolated and lonely. Despite this, the women had a positive attitude to their problems, which may have positively increased their ability to cope. The findings also revealed the importance of a reciprocal influence between the woman and her environment, and that social support was crucial.

**Conclusions:**

Pelvic girdle pain may influence women’s lives for months and years after delivery. Health care professionals should appreciate and focus on the patient’s knowledge and skills. Understanding the daily experiences of women with pelvic girdle pain might help improve rehabilitation strategies for these patients.

## Background

Pelvic girdle pain (PGP) is defined as pain in the pelvic musculoskeletal system that does not derive from gynaecological and urological disorders [1]. Whereas low back pain is defined as pain between the twelfth rib and the gluteal fold, PGP is defined as pain between the posterior iliac crest and the gluteal fold, particularly in the region around the sacroiliac joints and the symphysis pubis [1]. Even though the prevalence rates of pregnancy-related PGP vary depending on the criteria used and mode of reporting, it is estimated to be in the range of 24–50% during pregnancy [2, 3]. After delivery, severe pain and disability remain in 3–7% of women [1, 3, 4]. Although the aetiology of PGP is unknown, possible underlying mechanisms are hormonal, biomechanical, inadequate motor control and stress on ligament structures [5]. PGP is regarded as pathological when a woman needs professional help to cope with her daily life activities and it is suggested that PGP deserves serious attention from both clinical and research perspectives [3].

PGP during pregnancy greatly affects a woman’s experience of her pregnancy, her roles in relationships and her social context [6, 7]. These women are struggling with enduring pain that disturbs most aspects of their lives [6], and the pain is perceived as an unpredictable and potentially disabling condition [8]. It may also prevent the women from looking forward to future pregnancies [9], and negatively affects the role of being a mother [6]. Furthermore, there appears to be a lack of knowledge and awareness of PGP and how to support pregnant women with PGP both in society at large and among caregivers and employers [9]. The interaction with health care professionals is crucial and health care professionals must listen to the women and give them support [10].

Whereas most women recover after delivery, a number of women continue living with disabling PGP [1, 3, 4]. Chronic musculoskeletal pain belongs to the medically unexplained disorders and the way health care professionals perceive the women’s pain and handle their illness may be of vital importance to a woman’s understanding of herself and for recovery [11]. Acknowledging women’s experiences of PGP after delivery may be important in optimizing the health and lifestyle of women with long-lasting PGP. In-depth knowledge about women with PGP after delivery and their experiences of living with the physical, psychological and social aspects of PGP is scarce. There is, to our knowledge, no qualitative interview study describing women’s experiences of living with persistent PGP.

Hence, the aim of this study was to explore how women with post-partum PGP experience living with PGP pain and its influence on their daily life, and the challenges they encounter concerning their physical, psychological and social function.

## Method

A phenomenological–hermeneutical approach with semi-structured interviews was chosen to obtain in-depth knowledge of women’s experience of living with PGP after delivery [12].

### Participants

A strategic selection procedure was chosen to check that our informants were suffering from PGP [13]. The inclusion criteria were women with PGP after delivery who had contacted the health care services in connection with their PGP problems, and who tested positive on clinical tests for PGP. The clinical tests were Posterior Pelvic Pain Provocation test, Distraction test, Patrick’s Faber test, palpation of the symphysis and the Active Straight Leg Raising test [1, 14]. A minimum of two out of five positive tests was needed to be included in the study. Exclusion criteria were women who did not test positive on two or more of the tests, or who could not be interviewed in Norwegian.

To obtain the number of informants desired, women from two physiotherapy clinics and one hospital department in Southern Norway were consecutively recruited from December 2011 to January 2012. Two physiotherapists recruited informants at the physiotherapy clinics, and a medical doctor, recruited participants at the hospital. All of the requested informants who fulfilled the inclusion criteria agreed to participate in the study.

### Data collection

Two pilot interviews were performed to validate the interview questions; however, no changes were required. After the pilot interviews, an additional five in-depth interviews were conducted.

To avoid additional travel for the women, the interviews were performed in connection with planned treatment. The women were interviewed in a quiet environment, and the interviews lasted for 40–60 minutes. To minimize discomfort, the informant was offered a comfortable chair and invited to change position by standing or lying on a bench or mat during the interview.

Demographic questions about age, civilian status, employment and education level, number of children and time since last delivery were posed during each interview. The interview questions focused on describing a normal day, the physical challenges the women experienced, how the challenges affected them and their environment, and how they imagined their future would be. The interviewer focused on active listening and providing time for silence and reflection [15]. All interviews were audiotaped and transcribed verbatim by the first author.

Five informants with different ages and a large variability of pain duration were included in the study (Table 1). Four were recruited from the physiotherapy clinics and one from the hospital. Two had completed high school, and the other three had college- or university-level education. Their employment statuses were: student, nurse, optician, disability pensioner, on maternity leave, on sick leave. They reported from no comorbidity to a number of diseases and complaints such as obesity, high blood pressure, shoulder pain, allergy, hypermobility, respiration problems, fractures and back surgery.

**Table 1:** **Informants’ demographics**

Informant	Number and/Age of children	Marital status	PGP debut
A	1/7 months	Cohabitant	24^th^ week of pregnancy
B	2/2.5 years and 4 months	Married	1: 17^th^ week of pregnancy
			2: after delivery
C	2/4 years and 1.5 year	Married	8^th^ week of pregnancy
D	1/11 months	Married	15^th^ week of pregnancy
E	2/22 years and 11 years	Married	1: in connection with delivery
			2: 29^th^ week of pregnancy (she said she was really not free from pain from one pregnancy to the other)

### Data analysis

The text was transcribed by the first author and analysed using a phenomenological–hermeneutical approach [12]. Data were imported into NVivo 9 [16] and analysed according to Lindseth and Norberg’s three steps: naïve reading, structural analysis and comprehensive understanding of the text [12].

Through “Naïve Reading”, the transcribed text was read several times in its entirety to form an understanding of the women’s experiences. To achieve this, the authors had to be open and let the text speak [12]. The naïve understanding of the text held in a phenomenological language was the first step in the analysis and was validated by a structural analysis. Following the naïve reading, the structural analysis allowed themes to be identified and formulated. Two of the authors (JE and LF) divided the text into meaning units – the smallest part of a text that can stand alone and be meaningful. The units were condensed and categorized for similarities and differences, and then placed into abstract sub-themes and main themes. The themes were validated against the naïve understanding, and the themes and sub-themes of the structural analysis were discussed until consensus was achieved. Finally, to form a comprehensive understanding of the text, main themes and sub-themes were reflected upon in relation to the research question, the study’s context, the literature and previous research. The text was re-read with an open mind and with the naïve understanding and validated themes in mind [12]. Using the authors’ previous understanding and through further critical reflection, deeper awareness and understanding were achieved.

### Ethical considerations

The study was approved by the Department of Physical Medicine and Rehabilitation at the regional hospital and two physiotherapy clinics in Southern Norway, and the Regional Committees for Medical and Health Research Ethics (Ref. no.: 2011/2009). The study was conducted in accordance with the Helsinki Declaration and the Health Research Act [17]. All potential informants were given verbal and written information by the first author before the interviews and written informed consent was obtained. The participants were offered the opportunity to meet with a psychiatric nurse after the interview to deal with questions that might arise as a consequence of the interview.

## Results

The authors’ first understanding of the text after naïve reading was that the women experienced their problems as very challenging physically, mentally and socially. The women’s lives were characterized by pain, problems with mobility and mental stress, and their health problems affected their family and friends. Despite this, they also expressed experiences of coping, and it seemed that they assumed a positive attitude towards their problems.

The structural analysis revealed three main themes characterizing issues influencing women’s daily life after delivery: 1) activity and pain, 2) lack of acknowledgement of pain and disability, and 3) changed roles.

### Activity and pain

Informants described how their lives involved taking care of their children, their family and their home. Some of them described days when they felt exhausted because their own disability was more than they could cope with. They talked about having little private time, painful movements, and lack of energy and social interaction. All of them felt a great need to relax, yet they endeavoured to stay active. Physical limitations and pain were all-encompassing, but some experienced improvement during treatment, including reduced pain and improved energy and sleep:

*In the beginning, when I was standing at the bassinette … I almost cried. Because I was sort of standing like this … (pointing). Yes, it was so painful … in my lower back, back there (pointing to the pelvic joints). So … no, it has been challenging.* (Informant A)

Doing housework was a challenge for all of them and informant E said, *“No, it’s not much I can do. Yes, I have almost always had a cleaning lady* …*”.* Others received support from their partner, relatives or friends regarding housework activities.

Appreciation of physical activity and a desire to stay active were prominent. All of the women wanted to improve their activity levels. Those who were previously very active commented on the reduction in physical activity level in their daily lives:

*Because I have not been able to … walk on these paths where you are supposed to slide down on your back or climb a little and … I have not been able to move around there …. So it has been mostly walking on asphalt and gravel.* (Informant D)

A majority of the women noted problems engaging in hobbies or social activities they had enjoyed previously. Fatigue and especially problems related to sitting restricted their social activities. Informant C sighed, “*It really affects my whole life*”.

Coping strategies such as good planning, delegation, redistribution of energy, adjusted activity to avoid pain and the ability to find solutions seemed to be crucial to manage their daily life:

*It’s going pretty well … I do it little by little, and then when I empty the dishwasher, I do it gradually, taking first from the bottom and then … the top so … When I am doing the dishes I try to do it little by little. I also do a little dusting now and then.* (Informant C)

Information and knowledge were perceived as important to being able to cope. However, some experienced a lack of information about the delivery and their condition in hospital: “*I have often wondered if they had done things differently, then I might not have had my pelvic problems. However, I did not feel they responded to my questions*” (Informant B).

Support from family, friends and health professionals was essential. Many of the women experienced great variability in the treatment given by chiropractors, manual therapists, naturopaths and physiotherapists. Treatments such as massage, exercises, stretching, advice and guidance were described favourably. The informants emphasized the importance of individualized and regular physiotherapy for recovery and to prevent deterioration of their condition.

### Lack of acknowledgment of pain and disability

The great psychological stress caused by pain and disability was prominent in the women’s responses. The women found it difficult psychologically to experience physical inadequacy and to accept that their bodies did not allow them to perform activities to the level they expected. PGP affected their mood, and some commented that they were discouraged. The women commented that it was difficult when their problems were not acknowledged:

*They should have understood that something might be wrong; for instance, they may have suggested that I could have talked to the physiotherapist before discharge … Just simple actions like that … showing that they understand that I actually had a problem.* (Informant B)

Some informants experienced a lack of understanding from their colleagues or employers. Some even described being victimized by the health care system or colleagues, and issues such as abuse of power and loneliness were mentioned.

Expectations for the post-natal period were not met. Everyone wanted to regain their physical fitness even if this was difficult. Informant C said, “*It does happen that one would just love to be more active when with the children* … *could have gone skiing and such* … *done other things*”.

Despite their mental strain, the women had a positive attitude. They described hopes for the future with further recovery, reduced pain and increased physical activity:

*My hope for the future is that I will become a store manager (laughs) … and that I will have lost weight and have become much more physically fit … and that I’m a good mum … and a good partner. These people are the most important to me, I want them to have a good time, and then it will be important to be in good shape.* (Informant A)

### Changed roles

Several informants noted that their partner’s role at home had changed, especially concerning housework and childcare. Most of the women said they were dependent on their partner’s support: “*Earlier, it has always been me who made dinner, but now* … *he makes dinner every second day. This reduces the time I have to spend standing in the kitchen. And* … *he’s just become really clever*” (Informant D).

One of the women admired her husband for being so supportive and for appreciating her positive attitude: “*I think it was tough for him* … *It’s obvious* … *You do not like to see someone you love be in pain*” (Informant A).

Another woman expressed how frustrating it must be for her partner to adjust to her slow speed. The relationship between the women and their partners had changed: “*You are not that loving and tender anymore, I just want to say* … *go away (laughs). Leave me alone. Mm-hmm. So it affects us, yes, it does*” (Informant C).

Some of them assumed that their children had not noticed any change in their approach, whereas others believed that their children were affected by reduced interaction and activity. The women were concerned about their children and spent as much time as possible with them. They thought that their mood and reduced physical function could have an impact.

Informant C admitted: *“When I am in a lot of pain I become grumpy … or more edgy. I do lose my temper with the children … if I am in pain … it is not OK”.*

Support from family and friends was important to the women. Partners, mothers, mothers-in-law and friends helped with the housework and care of the child. Informant C said: “*But my closest friends know what I need* … *In a way, they try to give me a comfortable seat on the sofa or such things. It’s very nice; that makes me happy*”.

## Discussion

Mothering while living with a chronic disease is experienced as a challenging balance between being a mother and coping with the chronic disease [18, 19]. Our study confirms previous research describing that living with PGP is characterized by both physical, psychological and social challenges. Previous findings also found an association between the presence of emotional distress and PGP six months after delivery [4]. These findings are confirmed in our study as the women found it difficult to accept a daily life with pain and limited physical activity, and this made some of the women feel discouraged, isolated and lonely. It has previously been reported that living with constant pain disturbs most aspects of pregnant women’s lives, and makes their daily life a struggle [6, 19].

Our study aimed to explore women’s experiences of living with PGP. Antonovsky’s salutogenetic theory focusing on coping abilities and activities that strengthen health was chosen. Antonovsky is concerned with the relationship between health, stress and coping, and his “sense of coherence” (SOC) theory [20] was used to understand how living with PGP may affect women’s health. SOC consists of three interacting components: seeing a life event as comprehensible, manageable and meaningful [20]. These three components may explain why some people remain healthy, or even experience improved health despite extremely stressful experiences, while others experience impaired health through much less stress. According to Antonovsky [20], illness is a natural part of living; however, a person’s generalized resource deficits are individual. Living with chronic PGP influences a woman’s daily life, and thus also her health condition.

Despite their described strain, the women in our study assumed a positive attitude and had hopes for improved health in the future. According to Antonovsky’s SOC, the main motivating element needed to understand and deal with a challenge is a person’s mental health and experience of comprehensiveness [20]. This might be explained by the fact that these women in contrast to disabled mothers in general were healthy until the pregnancy or delivery, and that the hope of regaining their previous health status was a motivation. In contrast to women with disability who faced a chronic condition [21] the women in our study experienced acknowledgement of their situation and provision of treatment to increase their health which may increase their SOC. In our study, the women’s active coping strategies to manage their daily lives, such as avoiding exaggerated activity levels, or finding out what might have caused their problems, might have positively influenced their health. These findings are supported by studies showing that the majority of women with PGP during and after pregnancy do not report catastrophizing [22]. Pain intensity, fear-avoidance beliefs and health-related quality of life were not linked to persistent PGP [23].

The women in our study described a previous lack of acknowledgement of their experienced pain from health professionals. These findings are supported by Elden et al. [9], who described a lack of knowledge and awareness of PGP, and lack of support of pregnant women with PGP both in society at large and among caregivers and employers. Our findings are also consistent with research correlating patients’ mental health with health workers’ attitudes and knowledge [10, 24]. Professionals should accept the patients’ descriptions of pain and disability and acknowledge their need for treatment. The women’s experience of being involved and believed may strongly influence their SOC by making their daily lives comprehensible, manageable and meaningful [20].

The importance of information, seeing a physiotherapist and regular treatment was emphasized, and most of the women who received individualized treatment reported improved health. Knowledge about a patient’s experiences of pain and movement challenges is central in providing adequate treatment [25, 26]. This is supported by previous studies reporting that information, guidance, individual therapeutic exercises and development of altered movement patterns positively improved the women’s coping with PGP [1, 7, 10, 27]. It has also been shown that once an explanation of pain was given, a new understanding developed with gradual improvement in coping skills [28]. These findings are in accordance with a recent study of musculoskeletal problems where the importance of personalized focus was emphasized among patients with persistent medically unexplained physical symptoms [29].

The women’s health problems affected their partners as well as their children, family and friends, and the women experienced changed roles. Their partners, friends and family had become more supportive and took more responsibility for childcare, meals and other housework. According to Antonovsky [20], family, friends and the social environment affect SOC and health, and support from the environment is important to develop a strong SOC. Our findings are supported by Persson et al. [6] who found that PGP negatively affected the role of being a mother.

### Methodological strengths and weaknesses

Polit and Beck’s strategies for ensuring trustworthiness of qualitative studies were followed [13]. The first author’s background as a physiotherapist working with women with PGP for many years inspired her to do this study. However, to avoid entering into a therapeutic relationship and thus influencing the informants’ answers, the interviewer had no previous relationship with the informants before the interviews. Her role as a researcher was also emphasized in the beginning of each interview. To avoid bias, the data analysis process was performed in collaboration with the third author, who had no relationship with these informants [13].

The small number of five informants is, however, a limitation. Our intention was to obtain in-depth knowledge of women’s experiences with PGP post-partum, and with interviews lasting 40–60 minutes, a good insight into these women’s experiences was obtained. The sample consisted of five women, all with PGP, but with heterogeneity concerning age (range 25–47 years), duration of pain (range 1–22 years), time since delivery (range 7 months–22 years), co-morbidities (0–7) and employment (employed, student, on maternity leave, on sick leave and disability pensioner). Despite this diversity, their experiences of living with PGP revealed great similarities and the same themes emerged during the interviews, which strengthens the study’s credibility. Even though it seemed as if saturation was reached because new data did not provide new information, more informants might have given a broader picture of their experiences [13].

The first author’s reflective log, verbatim transcription and documentation of the analysis steps strengthen the study [13], together with the informants’ ability to give feedback on the interview text, even though no informants used this opportunity. The use of open questions was also a strength because some of the informants brought up unexpected topics based on their experiences with delivery such as lack of information after delivery, or positive or negative responses to previous treatment. All the participating women were in a relationship; hence, the absence of single mothers should be taken into consideration when considering the results of this study. Still, the study’s qualitative focus on women’s experiences makes it an important contribution to the limited research in this field.

## Conclusions

This study shows that PGP may influence women’s lives for months and years after delivery. Emotions such as discouragement, isolation and loneliness were a part of a daily life with pain and limited physical activity. Our findings imply that professionals should listen to and acknowledge patients’ descriptions of pain and disability. The importance of receiving information and individualized physiotherapy was emphasized as being important in coping with the situation. Understanding the daily experiences of women with PGP might help to improve rehabilitation strategies for these patients, and is crucial in policy-making, medical practice and in forming a better public health approach to women’s health.

## Abbreviations

PGP: Pelvic girdle pain
SOC: Sense of coherence.
